# Integration of *Immunome* With Disease-Gene Network Reveals Common Cellular Mechanisms Between IMIDs and Drug Repurposing Strategies

**DOI:** 10.3389/fimmu.2021.669400

**Published:** 2021-05-24

**Authors:** Abhinandan Devaprasad, Timothy R. D. J. Radstake, Aridaman Pandit

**Affiliations:** ^1^ Division Internal Medicine and Dermatology, University Medical Center Utrecht, Utrecht, Netherlands; ^2^ Center for Translational Immunology, University Medical Center Utrecht, Utrecht, Netherlands

**Keywords:** IMID (immune-mediated inflammatory diseases), disease-associated cells, disease-associated genes, drug repurposing, machine learning, *immunome*

## Abstract

**Objective:**

Development and progression of immune-mediated inflammatory diseases (IMIDs) involve intricate dysregulation of the disease-associated genes (DAGs) and their expressing immune cells. Identifying the crucial disease-associated cells (DACs) in IMIDs has been challenging due to the underlying complex molecular mechanism.

**Methods:**

Using transcriptome profiles of 40 different immune cells, unsupervised machine learning, and disease-gene networks, we constructed the Disease-gene IMmune cell Expression (DIME) network and identified top DACs and DAGs of 12 phenotypically different IMIDs. We compared the DIME networks of IMIDs to identify common pathways between them. We used the common pathways and publicly available drug-gene network to identify promising drug repurposing targets.

**Results:**

We found CD4^+^Treg, CD4^+^Th1, and NK cells as top DACs in inflammatory arthritis such as ankylosing spondylitis (AS), psoriatic arthritis, and rheumatoid arthritis (RA); neutrophils, granulocytes, and BDCA1^+^CD14^+^ cells in systemic lupus erythematosus and systemic scleroderma; ILC2, CD4^+^Th1, CD4^+^Treg, and NK cells in the inflammatory bowel diseases (IBDs). We identified lymphoid cells (CD4^+^Th1, CD4^+^Treg, and NK) and their associated pathways to be important in HLA-B27 type diseases (psoriasis, AS, and IBDs) and in primary-joint-inflammation-based inflammatory arthritis (AS and RA). Based on the common cellular mechanisms, we identified lifitegrast as a potential drug repurposing candidate for Crohn’s disease and other IMIDs.

**Conclusions:**

Existing methods are inadequate in capturing the intricate involvement of the crucial genes and cell types essential to IMIDs. Our approach identified the key DACs, DAGs, common mechanisms between IMIDs, and proposed potential drug repurposing targets using the DIME network. To extend our method to other diseases, we built the DIME tool (https://bitbucket.org/systemsimmunology/dime/) to help scientists uncover the etiology of complex and rare diseases to further drug development by better-determining drug targets, thereby mitigating the risk of failure in late clinical development.

## Introduction

Genetic and epigenetic heterogeneity plays a significant role in the development and progression of complex diseases. The past two decades have seen a major surge in studies that characterize genes and loci associated with diseases (1). The use of high-throughput omics technology and functional screenings have boosted our knowledge about genetic, epigenetic, and metabolic factors underlying complex diseases (1). As a result of these genetic and epigenetic screenings, we now know that most complex diseases and genes/loci have a many-to-many relationship. Meaning that complex diseases are linked to many different genes, and a gene/loci might be associated with several diseases (2). Thus, it is essential to identify and characterize these disease-associated genes (DAGs) to understand diseases better and develop therapy accordingly.

Extensive high-throughput screening studies and multi-omics have helped in the identification of DAGs. However, in most studies, DAGs were identified using bulk tissue or whole blood, a caveat since each gene’s expression is known to vary between tissues and cell types (3, 4). Thus, bulk tissue- or blood-based studies on DAGs do not consider the role of different cells and tissues in disease biology. To improve the understanding and molecular basis of complex diseases, a large number of research groups and consortiums have started to functionally identify disease-associated cells (DACs) or tissue types (3–7). The Genotype-Tissue Expression (GTEx) is one such valuable project, which maps gene expression profiles of 54 different human tissue types and the corresponding expression quantitative trait loci (eQTLs) (5–7). Furthermore, the growth of single-cell technologies propelled our understanding of diseases and helped in identifying DACs for complex conditions, including cancer (8), Alzheimer’s (9), rheumatoid arthritis (10), among others. Among these studies, the role of immune cells has been central to disease etiology and progression.

The immune system plays a vital role in developing and progressing immune-mediated and non-immune mediated chronic diseases. Many association and functional studies have shown that immune cells express multiple DAGs, and perturbing these DAGs can modulate immune cell functions (11). However, very few studies have explored the impact of DAGs on specific cell types and even fewer on immune cells, many of which focus on a limited number of cell subsets (12–16). Recently, Schmiedel et al. studied the effect of genetic variants on gene expression in 13 different immune cell types (17). However, this study primarily focused on analyzing genetic variants and their impact on a total of 13 immune cell types: monocytes (classical and non-classical), NK cells, naïve B-cells, and nine sub-populations of T-cells. The study identified several genetic variants to have a role in specific immune cell subsets in autoimmune disorders. For example, the modulatory effects of the variant rs12936231 in asthma and other autoimmune diseases are seen in lymphoid rather than myeloid subsets as previously described (17). Such new insights into specific immune cells’ role led us to believe that specific immune cells and their DAGs remain poorly understood even in immune disorders.

The immune-mediated inflammatory diseases (IMIDs) are complex among the immune disorders, involving several immune cells. For example, in rheumatoid arthritis, the immune cells such as B-cells, T-cells, macrophages, mast cells, dendritic cells, and NK cells play a significant role (18). However, the exact mechanism of these cell types remains unknown. Insights on the precise mechanism of action are crucial for developing successful therapies, which becomes particularly challenging for IMIDs due to several cell types involved. The massive undertaking of GWAS has enabled the mapping of some of the molecular mechanisms of the IMIDs (19–22). However, further research is required to understand the etiology of IMIDs taking into account the several different immune cells at play and the contributing DAGs for each immune cell type. By identifying the critical immune cells and their mechanism, we would set a robust rationale for identifying any mechanistic overlap between diseases and exploiting them to develop therapeutic strategies.

This study mapped the largest available and expert-curated disease-gene network (from the DisGeNet curated from 16 different databases) (23) on the most extensive *immunome* data comprising gene expression profiles of 40 different immune cell types, curated by us. We then used an unsupervised machine learning algorithm, the disease-gene network, and the *immunome* to create the Disease-gene IMmune cell Expression (DIME) network. Using this approach, we built a tool called DIME. Using DIME, we quantified the effects of 3957 DAGs on the *immunome* to identify DACs for 12 phenotypically different IMIDs. We used the DIME to (1) study the underlying cell-specific mechanisms (2); identify common DACs and their top-weighted DAGs between disease pairs (referred to as the common cell-gene network); and (3) identify drug repurposing targets using the common cell-gene network. The DIME is available as a user-friendly R tool (https://bitbucket.org/systemsimmunology/dime), to determine the top genes and cells associated with the disease of interest for (1): diseases from the DisGeNet (2), diseases from the EBI genome-wide association study (GWAS) catalog, or (3) custom set of genes defined by the user.

## Methods

### Transcriptome Data - *Immunome*

The transcriptome data consists of RNA-sequencing datasets of 40 different immune cell types curated using 316 samples from a total of 27 publicly available datasets (see **Supplementary Table S1** for list of GEO datasets and samples used). The 40 different immune cells cover the entire hematopoietic stem cell differentiation tree comprising nine progenitors, 19 lymphoid, and 12 myeloid cell types. The samples used here were manually curated considering only the unstimulated (except for monocyte-derived macrophages) immune cells that were sorted using Fluorescence-activated cell sorting (FACS) and were isolated from either blood, bone marrow, or cord blood from healthy donors.

All the selected datasets (**Supplementary Table S1**) were downloaded as FASTQ files using the fastq-dump tool from sratoolkit. The “split-files” option was given if the library type was paired-end sequencing. FASTQ files were then aligned to the reference genome (GRCH.Hg38.79) using the STAR aligner (24). The result is a SAM file, which was then converted into a sorted BAM file using the samtools program (25). These were then used to calculate the count of aligned reads using the HTSeq program (26) with the “intersection non-empty” option. HTSeq was run for all possible stranded mode options, the count file with the maximum counts was chosen as the respective count file for the sample.

The data was then filtered by removing all genes that had less than 20 read counts in 95 percent of the samples using R programming. The filtered data was then lane normalized using the “betweenLaneNormalization” function from the RUVSeq package (27). The RUVr method from RUVSeq was used to identify residual factors contributing to the batch effect. The resulting filtered, batch corrected, and normalized data had expression for 34,906 genes that were void of any observable batch effect. We calculated counts per million (CPM) for the filtered genes and used log2(CPM +1) as the gene expression measure. We then used the median gene expression for each cell type for the rest of the analysis. This processed, batch corrected, and normalized data of the 40 immune cells is referred to here as the *immunome*.

### Disease-Gene Network From DisGeNet

The disease-gene network from DisGeNet (23) was downloaded from the DisGeNet database (www.disgenet.org/downloads). All HLA associated genes were removed from the network; this was done to ensure that bias towards myeloid cells and B cells are removed since the HLA genes are primarily expressed by these cells. The resulting network was further filtered to include only those genes that were present in the *immunome*.

### IMID Disease-Gene Network

To study and identify the DACs of the IMIDs, we extracted the DAGs of 12 IMIDs extracted from the above DisGeNet. The IMID gene network for the 12 diseases comprised of 3579 DAGs. The 12 diseases that broadly represent the IMIDs in this study include: ankylosing spondylitis (CUI: C0038013), arthritis (CUI: C0003864), Crohn’s disease (CUI: C0010346), diabetes mellitus - non-insulin-dependent (CUI: C0011860), systemic lupus erythematosus (CUI: C0024141), multiple sclerosis (CUI: C0026769), psoriasis (CUI: C0033860), psoriatic arthritis (CUI: C0003872), rheumatoid arthritis (CUI: C0003873), Sjogren’s syndrome (CUI: C1527336), systemic scleroderma (CUI: C0036421), and ulcerative colitis (CUI: C0009324). CUI, used in DisGeNet, is the concept-unique-identifier for the disease term defined by the unified medical language system (28). The disease term arthritis (CUI: C0003864) comprises DAGs that pan over several arthropathies such as spondyloarthropathy, osteoarthritis, gout, allergic arthritis, etc., that fall under the broad arthritis MeSH term.

### Identification of Top DAC and DAG Using Machine Learning

Briefly, we used an unsupervised machine learning algorithm called non-negative matrix factorization (NMF) to map the disease-gene network to the *immunome* and identify the top DACs and DAGs of the 12 IMIDs. The NMF algorithm clusters the input gene expression data into ‘k’ clusters, such that the DAGs of a cluster are expressed by the DACs of the same cluster, thus forming DAC-DAG pairs in each cluster (29). We used the coefficients and weights identified by the NMF algorithm as the DAC and DAG scores, respectively. The scores were scaled between 0 and 1, with 1 being the highest score. Those in the top 25 percentile of the scores were regarded as the top DACs and DAGs, respectively. We calculated the Frobenius norm for each cluster to weigh and rank the clusters; the rank 1 cluster is the top cluster having the highest Frobenius norm value. The top cluster comprises the DAC-DAG pair that maximally captures/represents the input gene expression matrix. Using the top DAC-DAG pairs of all clusters, we constructed the Disease-gene IMmune cell Expression (DIME) network for the 12 IMIDs. Detailed description of the DIME method is as follows.

#### Mapping Disease-Gene Network to *Immunome* Data

For a given disease *D* and its DAG, we first extracted the corresponding *immunome* expression matrix (*X_D_*). *X_D_* comprised the gene expression of the DAG across the 40 cell types. *X_D_* was used as an input matrix for the NMF algorithm.

#### Using NMF to Cluster *X_D_* Into *k* Classes

We used the NMF package (30) in R and applied Brunet’s NMF algorithm (29) on *X_D_* to factor it into two matrices, namely *W_D_* and *H_D_* such that.

(1) $$X_{D}\approx W_{D}H_{D}$$

(2) $$W_{D}H_{D}=\left[\begin{array}{cccc} | & | & | & |\\ w_{D}^{1} & w_{D}^{2} & \ldots & w_{D}^{k}\\ | & | & | & | \end{array}\right]\left[\begin{array}{ccc} - & h_{D}^{1} & -\\ - & h_{D}^{2} & -\\ - & \vdots & -\\ - & h_{D}^{k} & - \end{array}\right]$$

(3) $$W_{D}H_{D}=\Sigma_{i=1}^{k}\;w_{D}^{i}\;h_{D}^{i};\;i\;\in \{1,\ldots ,k\}$$

Where *W_D_* and *H_D_* are the basis and coefficient matrices computed by NMF. Here, *k* is the number of classes/clusters that splits the data such that it satisfies the above NMF equations. The *W_D_* matrix comprises the weights of the DAGs across the *k* clusters (in each column), and the *H_D_* matrix comprises the weights of the cells in the corresponding *k* clusters (in each row). We used Brunet’s method to identify the ideal *k* value using the cophenetic correlation coefficient (29).

#### Identifying the Top DAG and DAC From *W_D_* and *H_D_*

The NMF algorithm clusters the data into *k* clusters (as shown in Equations 2 and 3) such that, in each cluster ‘*i*’, where *i* ∈ (1, …, *k*), the genes that have high values in $w_{D}^{i}$ are constitutively expressed by the cells that have high values in $h_{D}^{i}$. Where, $w_{D}^{i}$ is the *i* ^th^ column of *W_D_* and $h_{D}^{i}$ is the *i* ^th^ row of *H_D_*. We used the scaled (between 0 and 1) values of $h_{D}^{i}$ and $w_{D}^{i}$ as the DAC and DAG scores, respectively. For each cluster *i*, we chose the DACs and DAGs that were in the top 25^th^ percentile range of their DAC and DAG scores, respectively. These filtered DACs and DAGs are regarded as the top DACs and DAGs, respectively. The top DACs and DAGs were extracted for all clusters of *i* ∈ (1, …, *k*). The DIME network was constructed using the top DAC-DAG pairs from all clusters.

#### Identifying the Top Cluster

We then identified the largest weighted cluster (referred to as the top cluster) among the *k* clusters identified by the NMF. That is, the subset of DACs and DAGs of *X_D_* that can capture most of its expression pattern. We did this by calculating the Frobenius norm of each $w_{D}^{i}h_{D}^{i}$ for all values of *i* ∈ (1, …, *k*) from Equation 3. We then identified the top cluster for which ${\left||w_{D}^{i}h_{D}^{i}|\right|}_{F}$ is the maximum. This can be represented as:

(4) $$top\;cluster\;=\text{argmax}\left({\left||w_{D}^{i}h_{D}^{i}|\right|}_{F}\right);\;i\in \{1,\ldots k\}$$

Where the top cluster represents that which maximally captures/represents the expression matrix *X_D_*. Thus, the top cluster is the rank 1 cluster of DIME. Subsequent ranks are the next highest weighted clusters.

#### Evaluating the Consistency of Top DACs and DAGs Identified by DIME

To check the consistency of the results from DIME, we performed 1000 jackknife simulations for each of the 12 IMIDs. For each simulation of each disease, we ran DIME with 70% random subsampling of either the DACs or the DAGs. And in each simulation, we identified the top cluster and the top DACs when DAGs were subsampled and vice versa. We compared the consistency of the top DACs (**Supplementary Figure S2**) and the top 10 DAGs (**Supplementary Figure S3**) identified by the original DIME run (100% of the sample) against the 1000 simulations. We computed the Pearson correlation coefficient between the DAC/DAG score of the top cluster of the original run to the number of times the DAC/DAG was found as the top DAC/DAG in the top cluster of the 1000 simulations. We used the p-value from the Pearson correlation test to state the significance of correlation and thus the statistical significance of the top DAC/DAG of the top cluster.

### The Common Cell-Gene Network Between Diseases

To identify the common cell-gene network between two diseases, we looked at their overlapping DAC-DAG pairs in their corresponding DIME networks. We refer to the overlapping DAC-DAG pairs as the common cell-gene network between the two diseases. We then used the Jaccard index (JI) to measure the overlap between the two diseases and Fisher’s exact test (FET) to obtain a confidence p-value for the given overlap.

### Integrating Drug-Gene Network

We extracted the drug-gene target network from (1) DGIdb with the filter set to contain CHEMBL interactions of the drugs approved by the food and drug administration (FDA) of USA (31) (2); all drug-gene of CLUE database (32) and (3); all drug-gene of hPDI (33). The genes with drugs associated with them are labeled in the common cell-gene networks to highlight drugability (**Figures 6C–E**).

### Statistical Analysis

We performed 1000 jackknife simulations to assess the consistency of the results from the DIME (Supplementary Methods and **Supplementary Figures S2–S4**). We used the Pearson correlation coefficient to measure the significance of the jackknife simulations compared to the original run (**Supplementary Figure S4**).

## Results

### The Disease-Gene Network of the 12 IMIDs Reveal Several Common DAGs

**Figure 1:**
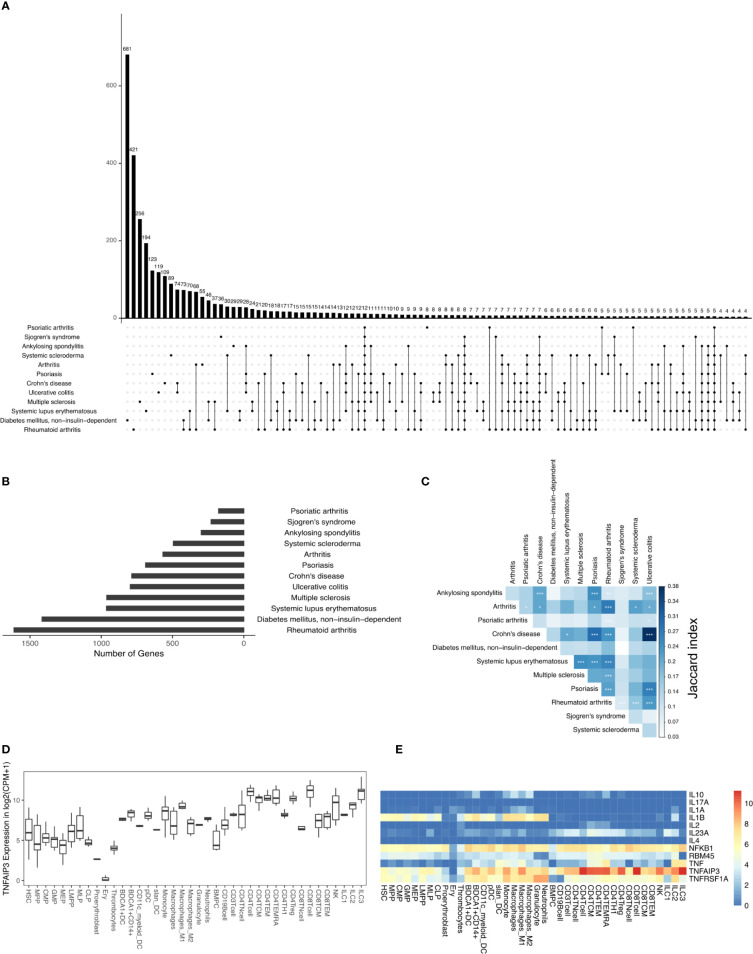
DAGs of IMIDs: **(A)** intersection of DAGs for all comparisons of IMIDs. Comparisons are shown only for those diseases that have at least one intersecting DAG between them. **(B)** Barplot represents the number of DAGs in each IMID. **(C)** Heatmap depicting Jaccard index and Fisher exact test (FET) p-value calculated for each IMID comparison. Fisher exact test (FET) p-value denoted by * (***≤ 0.001, **≤ 0.01 and *≤ 0.05). **(D)** Gene expression of TNFAIP3. **(E)** Heatmap depicting gene expression of the 12 genes common to all 12 IMIDs. Gene expression values in log2(CPM+1). CPM denotes counts per million.

In this study, we analyzed the DAGs of 12 different types of IMIDs that broadly include inflammatory arthropathies, spondyloarthropathies, rheumatic diseases, systemic IMIDs, and inflammatory bowel diseases (IBDs). And specifically, the 12 different IMIDs studied here are ankylosing spondylitis (298 DAGs), arthritis (567 DAGs), Crohn’s disease (786 DAGs), diabetes mellitus - non-insulin-dependent (1415 DAGs), systemic lupus erythematosus (963 DAGs), multiple sclerosis (961 DAGs), psoriasis (689 DAGs), psoriatic arthritis (177 DAGs), rheumatoid arthritis (1612 DAGs), Sjogren’s syndrome (229 DAGs), systemic scleroderma (494 DAGs), and ulcerative colitis (796 DAGs) (**Figures 1A, B** **)**. The 12 IMIDs had a total of 3957 DAGs. Among these, several genes were linked to several IMIDs; for example, 74 DAGs were linked to only Crohn’s disease (CD) and ulcerative colitis (UC), both IBDs. Calculating the Jaccard index and Fisher’s exact test (FET) on all the overlapping DAGs between all IMIDs revealed that CD and UC had the highest significant overlap (**Figure 1C**). Interestingly, genes associated with CD had significant overlap (FET p-value ≤ 0.05) with all diseases except psoriatic arthritis and non-insulin-dependent diabetes mellitus. Rheumatoid arthritis (RA) had a significant overlap of DAGs with all IMIDs except non-insulin-dependent diabetes mellitus. However, non-insulin-dependent diabetes mellitus did not have a significant overlap of DAGs with any of the IMIDs. Arthritis, psoriasis, CD, and RA had a significant overlap of DAGs between each other. We found 12 DAGs that were associated with all the 12 IMIDs (**Figures 1A, E** **)**. These DAGs were related to processes typically associated with inflammation, such as cytokine signaling (GO:0001817; GO:0019221), regulation of inflammatory response (GO:0050727), and regulation of interleukin-6 (GO:0032675; GO:0032635). We further explored these DAGs in the *immunome* and found the expression of TNFAIP3 to be the highest in CD8^+^ T-cells, ILC3 and CD4^+^ T-cells (**Figures 1D, E** **)**. Likewise, IL1B was expressed by myeloid and progenitor cells; TNF was expressed by lymphoid and myeloid cells. Overall, specific myeloid and lymphoid cells specifically expressed some of the 12 genes linked to all the 12 IMIDs. Such cell-specific expression of the DAGs led us to question the immune cell types and genes essential for the 12 IMIDs. Hence, we used the DIME on the 12 IMIDs to identify their top DACs and DAGs. Briefly, DIME uses the *immunome*, input disease-gene network, and an unsupervised machine learning algorithm (NMF) to determine the clusters of top DACs and DAGs.

### Inflammatory Arthritis Is Driven by CD4^+^ Treg, CD4^+^ Th1, and NK Cells

Inflammatory arthritis is an autoimmune disorder characterized by joint inflammation. And joint inflammation is the primary clinical feature observed in inflammatory arthritis types such as ankylosing spondylitis (AS) and RA. However, in other inflammatory arthritis types, such as psoriatic arthritis, inflammation is present in both the skin and joints. Interestingly, AS and psoriatic arthritis are both seronegative spondyloarthropathies (negative for rheumatoid factor and auto nuclear antibodies) characterized by enthesitis and predominant HLA-B27 genotype (34, 35). Such shared clinical features led us to question if the inflammatory arthritis types shared molecular mechanisms. So, we performed DIME on the different types of inflammatory arthritis to identify the critical DACs and DAGs and compare the molecular mechanism shared between them. As a reference, we used the broader arthritis disease term that encompassed several different kinds of arthropathies (including inflammatory arthritis).

**Figure 2:**
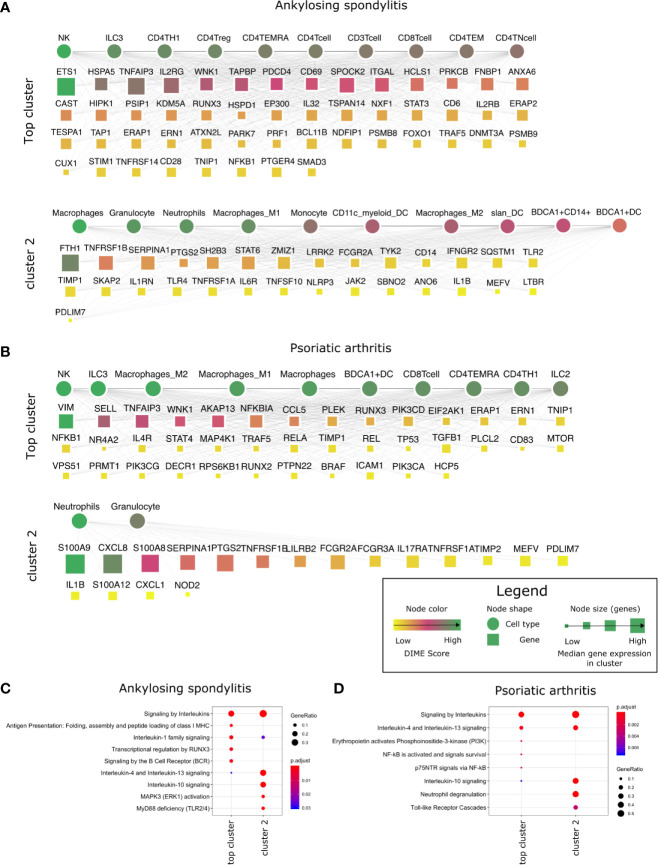
Top DACs and DAGs of inflammatory arthritis: DIME network of **(A)** ankylosing spondylitis and **(B)** psoriatic arthritis. The DIME network shows top 25 percentile DACs and DAGs. Square nodes represent genes and circular nodes represent cell types, the color scheme signify the DIME score (higher score signify importance of the node in the cluster) and the node size represent the median gene expression of the gene in the given cluster. Edges in each cluster signify the relationship between the cell types and genes as identified by DIME, where the cell types in the cluster express the genes of the same cluster. To aid visualization, the DAGs in the network is pruned based on the DIME score (top 50 DAGs if present) and gene expression (> 5 median gene expression in the corresponding cluster’s cell types). Pathway enrichment analysis of the top 25 percentile DAGs of **(C)** ankylosing spondylitis and **(D)** psoriatic arthritis.

**Figure 3:**
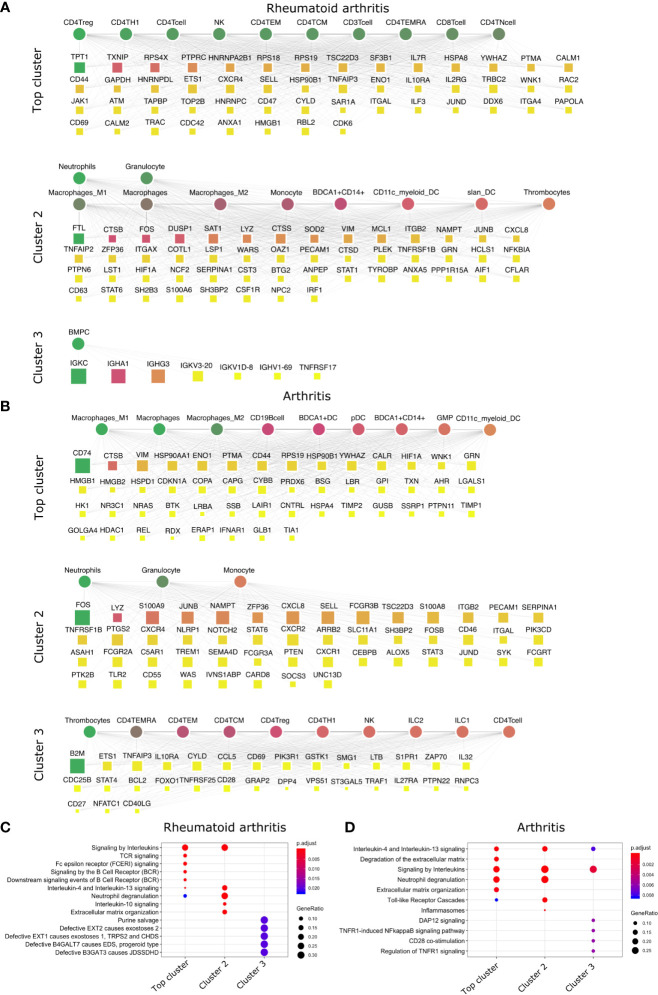
Top DACs and DAGs of inflammatory arthritis: DIME network of **(A)** rheumatoid arthritis and **(B)** arthritis. Pathway enrichment analysis of the top 25 percentile DAGs of **(C)** rheumatoid arthritis and **(D)** arthritis. See **Figure 2** legend for network description.

**Figure 4:**
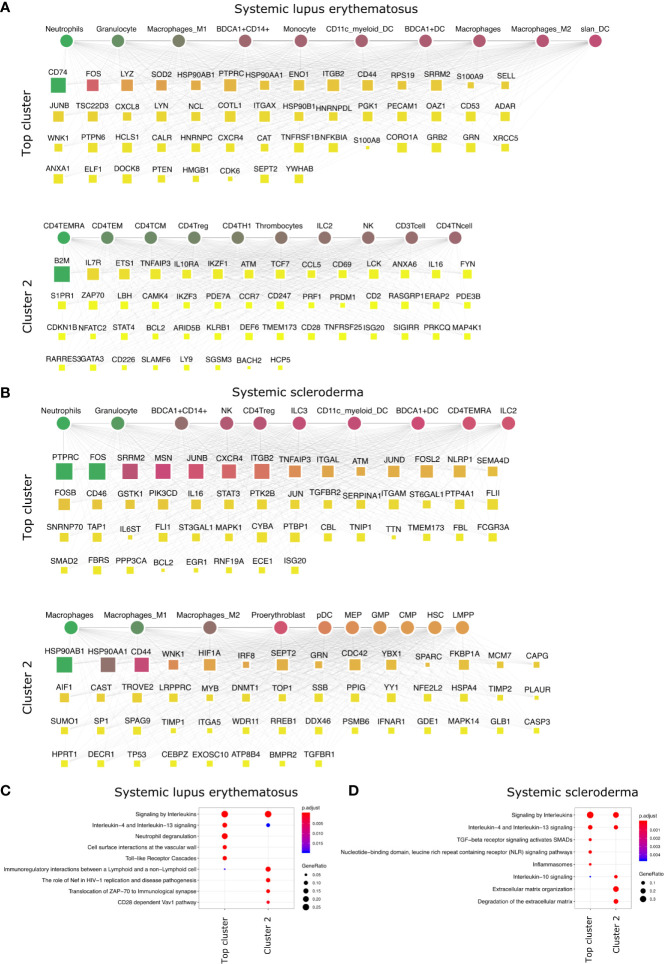
Top DACs and DAGs of systemic diseases: DIME network of **(A)** SLE and **(B)** arthritis. Pathway enrichment analysis of the top 25 percentile DAGs of **(C)** SLE and **(D)** SSc. See **Figure 2** legend for network description.

**Figure 5:**
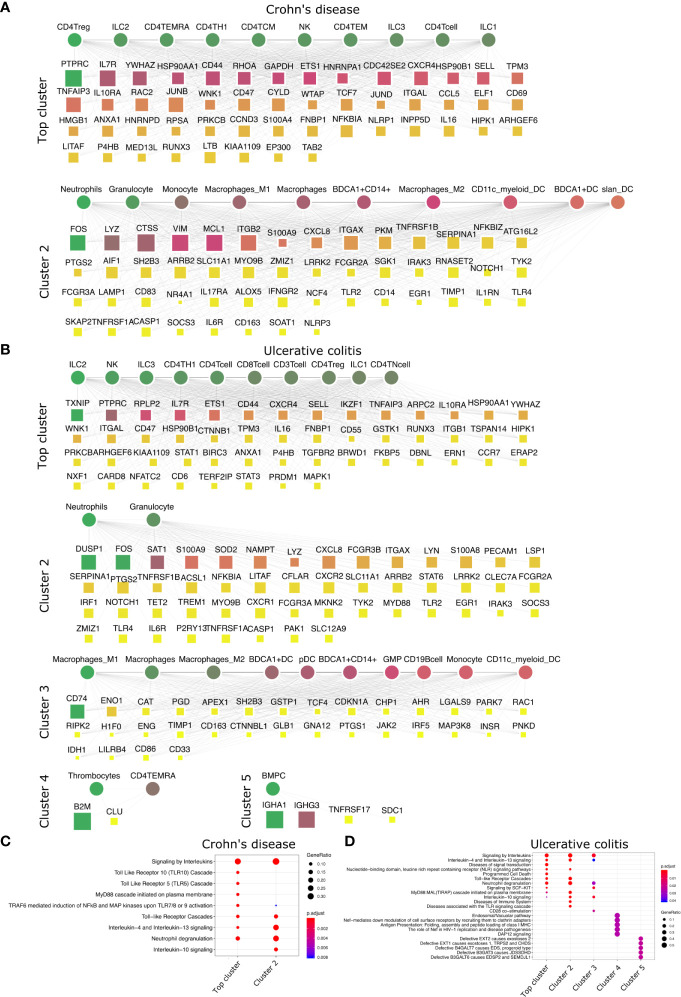
Top DACs and DAGs of IBDs: DIME network of **(A)** Crohn’s disease and **(B)** ulcerative colitis. Pathway enrichment analysis of the top 25 percentile DAGs of **(C)** Crohn’s disease and **(D)** ulcerative colitis. See **Figure 2** legend for network description.

In the DIME analysis, the clusters of the DIME network (**Figures 2**–**5**) are ordered based on the Frobenius norm. Cluster with the highest Frobenius norm represents the most crucial cluster and is designated as the top cluster comprising the most crucial DACs and DAGs. In each cluster, the DACs and DAGs are ordered based on the DIME score, high scoring nodes signify higher importance. The DIME analysis of AS revealed lymphoid cells such as NK cells, ILC3, CD4^+^ T-cells (Th1, Treg, TEMRA) as the top DACs in the top cluster (**Figure 2A**). The top cluster’s top DAGs comprised ETS1, HSPA5, TNFAIP3, IL2RG, WNK1, etc. that were associated with pathways such as interleukin signaling, antigen presentation, regulation of RUNX3, and BCR signaling (**Figure 2C**). We find RUNX3 expression highest in the NK cells, followed by CD8^+^ T-cells and Th1 cells (**Supplementary Figure S1A**). The exact role of RUNX3 in AS is unclear and possibly involves regulation, differentiation, and activation of Th1 and T-bet cells (36). Further research is required to establish the exact role of RUNX3 in AS and the above-identified lymphoid cell subsets. In the second cluster, the top DACs included myeloid cells, and the top DAGs were associated with pathways such as interleukin (IL-4, IL-10, IL-13) signaling, MAPK3 activation, and MyD88 (**Figures 2A, C** **)**. Thus, the key DACs of AS are diverse, as reported in the literature (37). However, according to DIME, the top DACs were NK cells, ILC3, CD4^+^ T-cells (Th1, Treg, TEMRA).

The DIME analysis of psoriatic arthritis revealed lymphoid cells such as NK cells, ILC3, T-cells (CD8^+^, TEMRA, Th1), ILC2 and myeloid cells like the macrophages and BDCA1^+^ DC as the top DACs in the top cluster (**Figure 2B**). Likewise, T-cells, NK cells, and antigen-presenting cells have played a crucial role in psoriatic arthritis pathology (38). The top cluster’s top DAGs were associated with interleukin (IL-4, IL-10, IL-13) signaling, PI3K, and NF-KB activation. (**Figure 2D**). Furthermore, the downstream genes of TNF-alpha such as TNFAIP3, TRAF5, NFKB1, and ICAM1 were top DAGs in the top cluster. Interestingly, TNF-alpha is a therapeutic target for psoriatic arthritis (39, 40), perhaps the downstream genes identified by DIME could also be explored as a therapeutic target for psoriatic arthritis. S100 calcium-binding proteins like S100A8 and S100A9 play a role in regulating inflammation in psoriatic arthritis (41). In the second cluster, we found the top DAGs included the S100 calcium-binding proteins, such as S100A9 and S100A8, which were highly expressed by neutrophils, granulocytes, monocytes, and dendritic cells (**Figure 2B** and **Supplementary Figure S1B**).

The crucial immune cells involved in RA are T-cells, B-cells, and APCs (42). While activation of CD4^+^ Th1 and impairment of CD4^+^ Tregs are essential for the pathology of rheumatoid arthritis (43), the DIME analysis of RA revealed several lymphoid cells such as CD4^+^ Tregs, CD4^+^ Th1, NK cells, etc., as the top DACs in the top cluster (**Figure 3A**). The top cluster’s top DAGs were associated with pathways such as interleukin, TCR, FCERI, and BCR signaling (**Figure 3C**). In the second cluster, the top DACs included myeloid cells, and the top DAGs were associated with pathways such as interleukin (IL-10, IL-13) signaling, neutrophil degranulation, and ECM organization (**Figures 3A, C** **)**. Activation, recruitment, and apoptosis of neutrophils are altered in RA, and under chronic inflammatory conditions, they release protease-rich granules (44).

The DIME analysis of the broader arthritis disease term revealed macrophages as the top DAC in the top cluster (**Figure 3B**). Macrophages play a central role in arthropathies, where they release cytokines and activate several immune cells such as T-cells, monocytes, neutrophils, and synovial fibroblasts. Besides, they are also the most abundant cells at the site of inflammation (45). The top DAGs of the top cluster were associated with pathways such as interleukin (IL-4, IL-13) signaling, extracellular matrix (ECM) related pathways, neutrophil degranulation, and toll-like receptor (TLR) cascades (**Figure 3D**). In the second cluster, the top DACs comprise neutrophils, granulocytes, and the top DAGs associated with pathways similar to the top cluster and included inflammasomes-related pathways (**Figures 3B, D** **)**.

In conclusion, using DIME, we found that in addition to the shared clinical features, the three inflammatory arthritis types also had a similar DAC profile comprising CD4^+^ Treg, CD4^+^ Th1, and NK cells as the top DACs, distinguishing them from the broader arthritis disease that showed macrophages as its top DAC. Perhaps these lymphoid cells contribute to inflammation in these arthropathies, and targeting them to reduce inflammation could be explored as a therapeutic strategy (46).

### Myeloid Cells Are Essential to Systemic IMIDs

We performed the DIME analysis on the systemic IMIDs such as systemic lupus erythematosus (SLE) and systemic scleroderma (SSc) (**Figure 4**). SLE and SSc are type I interferon-mediated systemic autoimmune diseases, that unlike RA, primarily affect not just the joints but also the skin, kidney, heart, and other organs (47). In SLE, the continuous IFN production by pDC and neutrophils leads to activation of monocytes, T-cells, and B-cells (48). The DIME analysis of SLE revealed the myeloid cells (granulocytes, macrophages, BDCA1^+^ CD14^+^, monocytes) as the top DACs in the top cluster (**Figure 4A**). The top DAGs in the top cluster were associated with interleukin signaling pathways (IL-4, IL-13), neutrophil degranulation, cell-surface interactions at the vascular wall, and the TLR cascades (**Figure 4C**). Incidentally, the neutrophils in SLE undergo spontaneous NETosis (a form of suicidal cell death), and this process is dependent on TLR signaling (48). Additionally, T-cells in SLE have altered cytokine production with higher IL6, IL7, and IL10 secretions (48). In the second cluster, we found the top DACs included CD4^+^ T-cells (TEMRA, TEM, TCM), and the top DACs were associated with pathways such as immunoregulatory interactions, Nef-associated factors (TNIP1, TNFAIP3), ZAP-70, VAV1 pathway (**Figures 4A, C** **)**. Nef-associated factors (TNIP1, TNFAIP3) have played a role in T-cell activation *via* TCR signaling in SLE (49).

The DIME analysis of SSc revealed myeloid cells (neutrophils, granulocytes, BDCA1^+^ CD14^+^ cells) and lymphoid cells (NK cells, CD4^+^ Treg, ILC2, and ILC3) as the top DACs in the top cluster (**Figure 4B**). The top DAGs in the top cluster were associated with interleukin signaling pathways (IL-4, IL-13), TGF beta signaling, NLR signaling, etc. (**Figure 4D**). In the second cluster, the top DACs included macrophages and the top DAGs were associated with pathways that included IL-10 signaling and degradation of ECM (**Figures 4B, D** **)**. As described in the review by Caam et al., several studies have shown neutrophils, macrophages, NK cells, and Tregs to play a role in the profibrotic events in SSc by the production of profibrotic cytokines such as TGF beta, IL-4, IL-10, IL-13, etc., thus corroborating our findings (50).

Thus, the top DACs of the systemic IMIDs comprised myeloid cells such as neutrophils, granulocytes, BDCA1^+^ CD14^+^ cells, CD11c^+^ myeloid cells, and BDCA1^+^ DC. Exploring the role of these cells, their corresponding DAGs and pathways in the systemic IMIDs may be useful for gaining mechanistic insights into disease and for successful therapeutic strategy. Exploring the role of neutrophils and their degranulation genes may serve as stronger targets in SLE, as neutrophil degranulation precedes type 1 interferon signature (often observed in SLE patients) (51–54). While the DACs of the SSc are diverse populations of myeloid and lymphoid cells, neutrophils are still an interesting candidate and exploring the role of TGF beta may shed light on the profibrotic events in SSc. Thus, based on the DIME analysis, attenuation of neutrophilic inflammation may be the key therapeutic strategy for the systemic IMIDs.

### IBDs Are Primarily Lymphoid Driven

We then looked at IMIDs that involve chronic inflammation of the digestive system, categorized as IBDs. The two primary forms of IBDs are CD and UC. CD is known to be driven by CD4^+^ Th1 cells, with a dominant Th1 cytokine profile leading to a pro-inflammatory effect (55). The DIME analysis of CD revealed lymphoid cells (CD4^+^ Treg, ILC2, CD4^+^ TEMRA, CD4^+^ Th1) as the top DACs in the top cluster (**Figure 5A**). The top DAGs of the top cluster were associated with pathways such as interleukin (IL-4, IL-10, IL-13) signaling, TLR (TLR-5, TLR-10) signaling, MyD88, and neutrophil degranulation (**Figure 5C**). In the second cluster, the top DACs included granulocytes, neutrophils, monocytes, macrophages, etc., and the top DAGs were associated with pathways such as interleukin signaling, neutrophil degranulation, and TLR cascades (**Figures 5A, C** **)**.

The T-cell profile of UC has been difficult to categorize due to discrepancies in its response among patients. However, there is evidence of Th2 cells, NK cells, macrophages, and neutrophils involved in the pathogenesis of UC (55). The DIME analysis of UC revealed lymphoid cells (ILC2, NK, ILC3, CD4^+^ Th1, etc.) as the DACs in the top cluster (**Figure 5B**). The top DAGs of the top cluster were associated with pathways such as interleukin (IL-4, IL-13) signaling, TLR cascades, NLR signaling, neutrophil degranulation, etc. (**Figure 5D**). In the second cluster, the top DACs included granulocytes, BDCA1^+^ CD14^+^ cells, etc. The top DAGs were associated with interleukin signaling pathways (IL-4, IL-10, IL-13), neutrophil degranulation, and TLR cascades (**Figures 5B, D** **)**. The DIME analysis of UC comprised 5 clusters in total, with myeloid cells in the third cluster, thrombocytes and TEMRA in the fourth cluster, and bone marrow plasma cells in the fifth cluster. This shows that the disease-gene network of UC involves genes that participate in many different cell types and are more complex to uncover their etiology. However, the highest weighted cluster signifies that the lymphoid cells may be the primary candidates for further analysis.

Thus, the top DACs of the IBDs include the ILC2, CD4^+^ Th1, CD4^+^ Treg, and the NK cells. The T-cells, innate lymphoid cells, and NK cells play an important role in the pathogenesis of IBDs (56–58), thus corroborating our findings. We hypothesize that Crohn’s disease is driven primarily by lymphoid inflammatory response and downregulating TLR signaling pathways could be a potential therapeutic strategy. The DAC profile of ulcerative colitis was found to be diverse, and would require a deeper analysis into the participation of the different cell types involved in their etiology.

### Statistically Significance of DIME Results

To evaluate DIME’s consistency, we performed 1000 Jackknife simulations with random subsampling of DAC/DAG. We re-identified the top DAC/DAG for all IMIDs (see **Supplementary Methods**). The jackknife simulations revealed that the top DACs identified across all clusters in the simulations (**Supplementary Figure S2A**) showed a similar pattern compared to top DACs identified in the original run (**Supplementary Figure S2C**). For the top DACs of the top cluster, the pattern from the simulations (**Supplementary Figure S2B**) was comparable to the original run’s DAC score (**Supplementary Figure S2D**). We used Pearson correlation to compare the pattern observed between the simulations and the original run. The Pearson correlation between the pattern observed in the simulated run (**Supplementary Figure S2B**) and the DAC scores of the original run for the top cluster revealed that the top DACs in the top cluster were significantly correlated (p-value ≤ 0.05) for all the IMIDs except ulcerative colitis (**Supplementary Figure S4**). This correlation shows that the top DACs of the top cluster identified by DIME is statistically significant for all IMIDs, except UC.

Likewise, we evaluated the consistency of the top DAGs. In all simulations, the top 10 DAGs of the original run’s top cluster were present as the top DAG in any of the simulated run clusters (**Supplementary Figure S3**). The Pearson correlation between the pattern observed in the simulated run and the DAG scores of the original run for the top cluster was significantly correlated for all the IMIDs (**Supplementary Figure S4B**). Thus, the top DAGs of the top cluster identified by DIME is statistically significant for all IMIDs.

### Why Are the top DACs of UC Insignificant?

In the case of UC, the top DACs were statistically insignificant from our 1000 jackknife simulations; the top DAGs, however, were significant (**Supplementary Figures S2**–**S4**). We found from 1000 simulations that the lymphoid cells identified by the original run (**Figure 5B**) were indeed present in the simulations. The myeloid cells were also part of the top DACs of the top cluster in the simulations (**Supplementary Figure S2B**). Furthermore, we found that the top cluster’s top DAGs included genes associated with neutrophil degranulation pathways and other myeloid cell-related pathways (**Figures 5B, D** **)**. Thus, owing to the non-convergence of NMF in accurately predicting the top DACs of the top cluster in UC. The top DACs of the top cluster of UC were ambiguous, as reported in the literature (55). From our simulations, we propose the inclusion of the myeloid cells in the top DACs of the top cluster in addition to the lymphoid cells previously identified (**Figure 5B**).

### Common Cell-Gene Networks Reveal Common Mechanisms Between IMIDs and Potential Drug Targets

**Figure 6:**
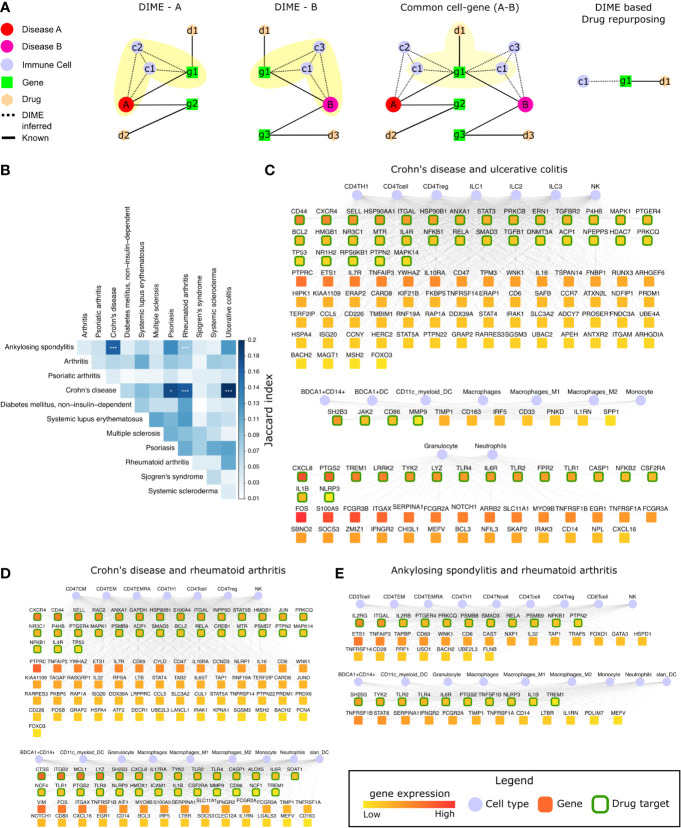
Common mechanisms between IMIDs. **(A)** Steps involved in DIME based drug repurposing using the common cell-gene network. **(B)** Jaccard index and FET calculated for the common cell-gene between two diseases for all disease comparisons. Fisher exact test (FET) p-value denoted by *(***≤ 0.001 and *≤ 0.05). The common cell-gene network of **(C)** Crohn’s disease and ulcerative colitis, **(D)** Crohn’s disease and rheumatoid arthritis, and **(E)** ankylosing spondylitis and rheumatoid arthritis. The DAG’s color is based on the median gene expression of the DAG in the corresponding DACs. DAGs that are drug targets have a green border, and the cells are shown in blue color.

The DIME analysis revealed that several top DAGs and their corresponding DACs were present in many IMIDs. For example, in many IMIDs, the gene FOS was present as top DAG in the cluster typically containing myeloid cells (granulocytes, neutrophils, and dendritic cells) as the top DACs. We found several genes, like FOS, that were present as the top DAG in the same top DAC cluster between different pairs of diseases. We refer to these top DACs and DAGs present between the two diseases as the common cell-gene network (represented schematically in **Figure 6A**). Using the common cell-gene network, we suggest that these diseases may have a similar mechanism of action. We could exploit such common mechanisms to gain mechanistic insights between diseases and identify drug repurposing targets. Hence, we integrated the publicly available drug-gene networks to identify and reinforce drug repurposing targets based on the common mechanisms (cell-gene networks) determined from the DIME analysis (**Figure 6A**).

To identify the common mechanisms across the 12 IMIDs, we identified the common cell-gene networks between all disease comparisons (**Figure 6B**). We then used the Jaccard index and FET to measure the extent and significance of the overlap in the common cell-gene networks between the disease pairs. Compared to the analysis that looked at all DAGs, which showed several diseases to be statistically significant in the overlap, the common cell-gene network overlap was restricted to fewer diseases (**Figure 1C** and **Figure 6B**).

The comparative analysis revealed that CD had statistically significant common cell-gene networks with several diseases such as, psoriasis, RA, and UC (**Figure 6B**). CD and UC’s common cell-gene network had the highest Jaccard index among all the IMIDs, both being IBDs with an aggressive T-cell response (55). CD and UC’s common cell-gene network revealed that the top DACs included the lymphoid cells such as CD4^+^ T-cell, CD4^+^ Th1, CD4^+^ Treg, ILC1, ILC2, ILC3, and NK cells in one cluster (**Figure 6C**). CD4^+^ Th1 and NK cells are known to be implicated in both CD and UC (55). The top DAGs (represented by green border) such as CD44, CXCR4, SELL, HSP90AA1 etc., were highly expressed by cells of the lymphoid cluster and were also drug targets (genes that are druggable or have drug targeting them in our drug-gene network). Thus, making them potential drug repurposing candidates for CD and UC. Other potentially interesting DAGs (that were not drug targets) included PTPRC, ETS1, IL7R, TNFAIP3, etc. Collectively, the DAGs in the lymphoid cluster were enriched in interleukin signaling pathways (IL-4 and IL-13), NLR signaling, etc. (**Supplementary Figure S5A**). The other clusters consisted of myeloid cells such as the granulocytes, dendritic cells, monocytes, and macrophages, among which dendritic cells have been crucial for regulating the T-cell responses in IBDs. The top DAGs, such as SH2B3, JAK2, CD86, MMP9, CXCL8, PTGS2, TREM1, LRRK2, TYK2, etc., were highly expressed by the myeloid cluster cells and were potential drug repurposing candidates. These DAGs were enriched in interleukin signaling pathways (IL-10), TLR signaling, ECM degradation, etc. (**Supplementary Figure S5A**).

We next explored the common cell-gene network of the two distinct IMIDs that belonged to different pathophysiology, namely CD and RA. The common cell-gene network of CD and RA revealed that the top DACs comprised of the lymphoid cells that included all CD4^+^ T-cells and NK cells in one cluster (**Figure 6D**). The top DAGs such as CXCR4, CD44, SELL, RAC2, ANXA1, etc., were highly expressed by this cluster’s cells and were potential drug repurposing candidates. These DAGs were enriched for pathways associated with interleukin, TLR, MyD88 signaling, etc. (**Supplementary Figure S5B**). The other cluster comprised myeloid cells such as granulocytes, dendritic cells, monocytes, and macrophages. The top DAGs such as CTSS, ITGB2, MCL1, LYZ, SH2B3, etc., were highly expressed by this cluster’s cells and were potential drug repurposing candidates. These DAGs were enriched for pathways associated with interleukin (IL-4, IL-13) signaling, neutrophil degranulation, etc. (**Supplementary Figure S5B**).

In addition to CD’s common cell-gene networks, we found a statistically significant common cell-gene network between the two inflammatory arthropathies that have joint pain as the primary feature, namely AS and RA. The common cell-gene network of AS and RA revealed that the top DACs comprised of the lymphoid cells that included all the T-cells and NK cells in one cluster (**Figure 6E**). The top DAGs such as IL2RG, ITGAL, IL2Rb, PTGER4, PRKCQ, etc., were highly expressed by this cluster’s cells and were potential drug repurposing candidates. These DAGs were enriched for interleukin (IL-1) signaling pathways, FCERI mediated NF−kB activation, TCR signaling, etc. The other clusters comprised myeloid cells such as granulocytes, dendritic cells, monocytes, and macrophages. The top DAGs, such as SH2B3, TYK2, TLR2, TLR4, IL6R, were highly expressed by this cluster’s cells and were potential drug repurposing candidates. These DAGs were enriched for interleukin signaling pathways (IL-4, IL-10, IL-13) (**Supplementary Figure S5C**).

Thus, using the common cell-gene networks, we could uncover the common mechanisms between the IMID pairs (**Figure 6** and **Supplementary Figure S5**) and use them to identify potential drug targets. This novel method of computational drug repurposing is a combination of target-based and mechanism-based drug repurposing strategies (59). We found several DAGs such as IL1B, IL6R, ITGAL, PTGS2, TYK2, NFKB1, NLRP3, PRKCQ, PTGER4, PTPN2, RELA, SH2B3, SMAD3, TLR2, TLR4, and TREM1, that were drug targets and present in all the common cell-gene networks (**Figures 6C, E** **)**. Among these DAGs, ITGAL was the only DAG that was a drug target and present as the top DAG of the top cluster (lymphoid cell cluster) in the DIME networks of CD, UC, AS, and RA. This is interesting as the lymphoid cells were identified as the top DAC of the top cluster for all of the above diseases. Using the drugs associated with these drug targets specifically for these diseases (CD, UC, AS, and RA) in therapy would require extensive experimental validation and clinical trials. Therefore, we explored (in the next section) the possibility of using some of these drug targets for repurposing based on existing studies and drugs that are already approved by the FDA. Thus, reinforcing and strengthening these targets and also the validity of our approach in identifying them.

### Common Cell-Gene Networks Reveal Drug Repurposing Targets

To explore and validate the drug targets for repurposing, we focused on the top DAGs of the statistically significant (FET p-value ≤ 0.05) common cell-gene networks of all IMIDs (**Figure 6B**). To identify drug targets that were targets of FDA-approved drugs, we used the drug-gene network of CHEMBL. We found several drug targets such as IL1B, IL6R, ITGAL, and TYK2 to be present in all the statistically significant common cell-gene networks (**Table 1**). Here, we explore the possibility of using these drug targets for repurposing based on their current use as a therapeutic target in certain IMIDs and suggest where they can be repurposed based on their presence in the common cell-gene networks of those IMIDs and others.

**Table 1:** The top DAGs and their FDA approved drug candidates identified from the DIME-based common cell-gene networks of the different IMIDs.

DAG/Drug target	Diseases	Drugs
IL1B	AS, CD, Psoriasis, RA, UC	canakinumab, rilonacept, anakinra
IL6R	AS, CD, Psoriasis, RA, UC	tocilizumab
ITGAL	AS, CD, Psoriasis, RA, UC	lifitegrast
TYK2	AS, CD, Psoriasis, RA, UC	tofacitinib citrate
PSMB9	AS, CD, Psoriasis, RA	bortezomib, carfilzomib, ixazomib citrate
DNMT3A	AS, CD, Psoriasis, UC	azacitidine, decitabine
HDAC7	AS, CD, Psoriasis, UC	belinostat, panobinostat lactate, romidepsin
JAK2	AS, CD, Psoriasis, UC	baricitinib, ruxolitinib phosphate, tofacitinib citrate
PTGS2	AS, CD, RA, UC	acetaminophen, aminosalicylate potassium, aminosalicylate sodium, aspirin, balsalazide disodium, bismuth subsalicylate, bromfenac sodium, carprofen, diclofenac, diclofenac epolamine, diclofenac potassium, diclofenac sodium, diflunisal, etodolac, etoricoxib, fenoprofen calcium, flurbiprofen, flurbiprofen sodium, ibuprofen, ibuprofen lysine, ibuprofen sodium, indomethacin, indomethacin sodium, ketoprofen, ketorolac tromethamine, meclofenamate sodium, meloxicam, mesalamine, nabumetone, naproxen, naproxen etemesil, naproxen sodium, nepafenac, olsalazine sodium, oxaprozin, oxaprozin potassium, piroxicam, sulfasalazine, sulindac, tolmetin sodium
BCL2	CD, Psoriasis, RA, UC	venetoclax
CXCR4	CD, Psoriasis, RA, UC	plerixafor
IL4R	CD, Psoriasis, RA, UC	dupilumab
IL17RA	CD, Psoriasis, RA	brodalumab
ITGB2	CD, Psoriasis, RA	lifitegrast
PSMD7	CD, Psoriasis, RA	bortezomib, carfilzomib, ixazomib citrate
CD86	CD, RA, UC	abatacept, belatacept
CSF2RA	CD, RA, UC	sargramostim
NR3C1	CD, RA, UC	alclometasone dipropionate, amcinonide, beclomethasone dipropionate, betamethasone, betamethasone acetate, betamethasone benzoate, betamethasone dipropionate, betamethasone sodium phosphate, betamethasone valerate, budesonide, ciclesonide, clobetasol propionate, clocortolone pivalate, cortisone acetate, deflazacort, desonide, desoximetasone, dexamethasone, dexamethasone acetate, dexamethasone sodium phosphate, diflorasone diacetate, difluprednate, flumethasone pivalate, flunisolide, fluocinonide, fluorometholone, fluorometholone acetate, fluprednisolone, flurandrenolide, fluticasone furoate, fluticasone propionate, halcinonide, hydrocortamate hydrochloride, hydrocortisone, hydrocortisone acetate, hydrocortisone butyrate, hydrocortisone cypionate, hydrocortisone probutate, hydrocortisone sodium phosphate, hydrocortisone sodium succinate, hydrocortisone valerate, loteprednol etabonate, medrysone, meprednisone, methylprednisolone, methylprednisolone acetate, methylprednisolone sodium succinate, mifepristone, mometasone furoate, paramethasone acetate, prednicarbate, prednisolone, prednisolone acetate, prednisolone sodium phosphate, prednisolone tebutate, prednisone, rimexolone, triamcinolone, triamcinolone acetonide, triamcinolone diacetate, triamcinolone hexacetonide
P4HB	CD, RA, UC	lomitapide mesylate
IL2RB, IL2RG	AS, RA	basiliximab, daclizumab
PSMB8	AS, RA	bortezomib, carfilzomib, ixazomib citrate
ALOX5	CD, RA	balsalazide disodium, meclofenamate sodium, mesalamine, olsalazine sodium, sulfasalazine, zileuton

Drug targets identified by us that are already in use for the different IMIDs include drugs that target IL1B, IL6, TYK2, and JAK2.IL1B was present in the common cell-gene network of AS, CD, psoriasis, RA, and UC, indicating a similar mechanism between these diseases (**Table 1**). Anti-IL1 therapy is used for psoriasis and RA (60–62), suggesting that it could be potentially used in IMIDs like AS, CD, and UC. Incidentally, preliminary studies indicate that anti-IL1 treatment has shown promising clinical response for treating AS, CD, and UC (63, 64). Anti-IL6 therapy (tocilizumab) showed a positive clinical response in a small group of patients in AS, CD, and RA, suggesting its application in other IMIDs like psoriasis and UC (65–67). However, anti-IL6 therapy had side effects in smaller studies on psoriasis and UC and must be explored carefully (68, 69). Tofacitinib, a TYK2 and JAK2 inhibitor developed for RA, is now making way to treatment options in other diseases such as, CD, UC, and psoriasis (70–73). Plerixafor (drug target: CXCR4) is a drug now used in cancer (lymphoma and multiple myeloma) after stem cell transplantation to initiate migration of stem cells in the bloodstream (74). This drug is now in clinical trials (NCT01413100) to be evaluated for use after autologous transplant in patients with SSc. We suggest extending such trials based on exploiting the CXCR4 mediated dysregulation of the immune system to other IMIDs like psoriasis, CD, RA, and UC.

Integrin based therapies (such as natalizumab and vedolizumab that targets ITGB2) are already used for CD (75). Exploring other integrin based therapies (such as Lifitegrast that targets ITGAL and ITGB2) for CD may be beneficial since ITGAL and ITGB2 are top DAGs in the DIME network and are also implicated in CD (76, 77). Lifitegrast could be a promising drug repurposing candidate for CD and perhaps for UC, AS, and RA. Since its target gene ITGAL, was the only top DAG of the top cluster (lymphoid cell cluster) that was also a drug target in the DIME networks of these diseases (**Figures 2**, **3**, **6C–E**). Thus, we propose lifitegrast as a novel drug repurposing candidate to be tested for CD, UC, AS, and RA.

## Discussion

Despite decades of experimental data, the knowledge of important cell types involved in the disease’s pathogenesis remains limited. To address this gap, we used the *immunome* comprising 40 immune cells, the disease-gene network, and computational methods to identify the important DACs and DAGs of the disease. The integration of these parts resulted in the novel mechanisms being captured by our method, using which we built a tool called the DIME. Here, we highlight the important DACs, DAGs, and common mechanisms captured using DIME for 12 phenotypically different IMIDs. Using DIME, the top DACs were found to be CD4^+^ Treg, CD4^+^ Th1, and NK cells in inflammatory arthritis (AS, PsA, and RA); neutrophils, granulocytes, and BDCA1^+^ CD14^+^ cells in SLE and SSc; ILC2, NK, CD4^+^ Th1, and CD4^+^ Treg in the IBDs.

Lymphoid cells such as CD4^+^ Th1, CD4^+^ Treg, and NK cells were the key players in inflammatory arthritis (AS, PsA, and RA) and IBD (CD and UC). These diseases have been reported to have an intricate cross-play of the above lymphoid cells, where the NK cells influence the differentiation of CD4^+^ Th cells into CD4^+^ Th1 and CD4^+^ Tregs; CD4^+^ Th1 plays a crucial role in the initiation of inflammation by cytokine production; the CD4^+^ Tregs are crucial for immune response modulation (78). Interestingly, the top DAGs of these diseases show pathways associated with the signaling of IL-4 and IL-13 that are crucial in this cross-play, thus corroborating DIME results.

Although we excluded HLA genes to prevent myeloid and B cell bias, the IMIDs associated with the HLA-B27, such as psoriasis, AS, and IBDs were found to have statistically significant common-cell gene networks. However, PsA (also associated with HLA-B27) is omitted here since it did not have a statistically significant common-cell gene network with any IMIDs (**Figure 6B**). Additionally, AS and RA, the two inflammatory arthritis with joint inflammation as the primary feature, also had a statistically significant common-cell gene network. Thus, the diseases with these shared clinical features also had common mechanisms as identified by DIME. The common mechanisms from these networks revealed several lymphoid and myeloid cells and their expressing DAGs. The lymphoid cells such as CD4^+^ Th1, CD4^+^ Treg, and NK were predominant in all the statistically significant common-cell gene networks, showing that these diseases were indeed mainly driven by the aggressive T-cell response (36–38, 55). Pathways such as interleukin (IL-4 and IL-13), TLR, TCR signaling, etc., was found to be enriched in the top DAGs of the common cell-gene networks of these IMIDs. Thus, the common cell-gene network revealed several common mechanisms between the diseases in accordance with the top DACs, DAGs, and their associated pathways.

We used the common mechanism from the common cell-gene network and the drug-gene networks to propose potential drug targets for repurposing. This novel computational drug repurposing strategy, a combination of target-based (literature drug-gene network) and mechanism-based (inferred from DIME), revealed several potential drug targets such as IL1B, IL6R, ITGAL, PTGS2, TYK2, NFKB1, NLRP3, PRKCQ, PTGER4, PTPN2, RELA, SH2B3, SMAD3, TLR2, TLR4, and TREM1. Further, we used these mechanism-based drug targets from DIME, and the FDA approved drug-gene network to propose several drug targets and their drugs that could expedite the drug repurposing process (**Table 1**). Thus, we were able to capture drug targets and their drugs currently being targeted or being explored for use in therapy for the IMIDs. We also found a few novel targets, such as the drug lifitegrast (used for dry eyes) for CD, UC, AS, and RA as an alternative to other integrin-based therapies already in use for CD. Lifitegrast is particularly interesting because it targets ITGAL, which was found to be important in the lymphoid cell cluster of CD, UC, AS, and RA. Thus, effectively targeting the exact mechanism. Perhaps the effect of lifitegrast could be used for down-regulating lymphoid cell-mediated inflammation in these diseases (79). Although Lifitegrast is currently available as an eye drop application and used to treat eye complications only, different formulations of this drug can be explored to treat CD, UC, AS, and RA. To the best of our knowledge, lifitegrast in the axis of ITGAL has not been investigated to treat CD, UC, AS, and RA. Thus, using DIME, we were able to propose a novel drug repurposing strategy from the analysis of the 12 IMIDs.

In conclusion, DIME helped identify (1) top DACs, DAGs of the IMIDs (2), Common mechanisms between the IMIDs, and (3) drug targets for repurposing. To enable DIME analysis for other diseases from the DisGeNet, the GWAS network, or a user-defined set of genes, we built the DIME tool as a user-friendly shinyapp. We believe that this tool will help scientists uncover the etiology of complex and rare diseases and facilitate drug development by better-determining drug targets, thereby mitigating the risk of failure in late clinical development.

## Data Availability Statement

The original contributions presented in the study are included inthe article/**Supplementary Material**. The DIME tool is available on https://bitbucket.org/systemsimmunology/dime. Further inquiries can bedirected to the corresponding author.

## Author Contributions

AD and AP were involved in the conception of the study. AD was involved in the data curation, visualization, and R shiny package development. AD and AP were involved in the data analysis and interpretation. AD and AP drafted the manuscript. TR helped in writing and revising the manuscript and discussions about clinical perspective. All authors contributed to the article and approved the submitted version.

## Funding

This project was supported by the Netherlands Organization for Scientific Research (NWO; Grant number 016.Veni.178.027).

## Conflict of Interest

The authors declare that the research was conducted in the absence of any commercial or financial relationships that could be construed as a potential conflict of interest.
