# Supplementary material for: Integration of Immunome With Disease-Gene Network Reveals Common Cellular Mechanisms Between IMIDs and Drug Repurposing Strategies
Source: Front Immunol. 2021 May 24;12:669400. doi: 10.3389/fimmu.2021.669400 (PMC8181425; doi:10.3389/fimmu.2021.669400)
Supplement: Supplementary file 1 [file DataSheet_1.pdf]

Supplementary Information

Integration of immunome with disease-gene network reveals common cellular mechanisms between IMIDs and drug repurposing strategies

Abhinandan Devaprasad[1,2], Timothy RDJ Radstake[1,2], Aridaman Pandit[1,2*]

[1] Division Internal Medicine and Dermatology, University Medical Center Utrecht, Utrecht, The Netherlands

[2] Center for Translational Immunology, University Medical Center Utrecht, Utrecht, The Netherlands

Supplementary Figures

A

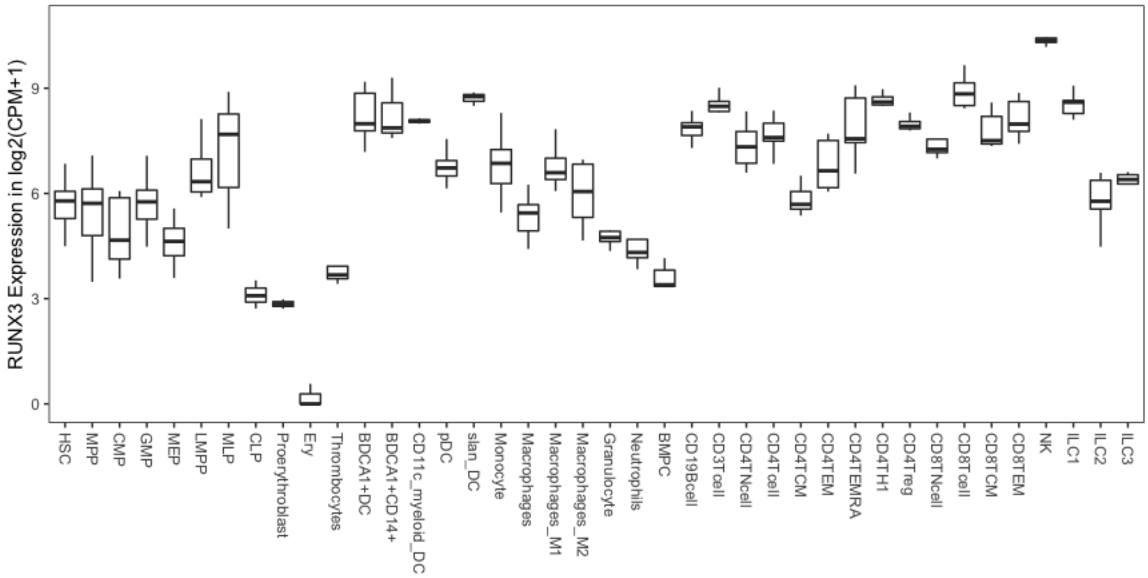

B

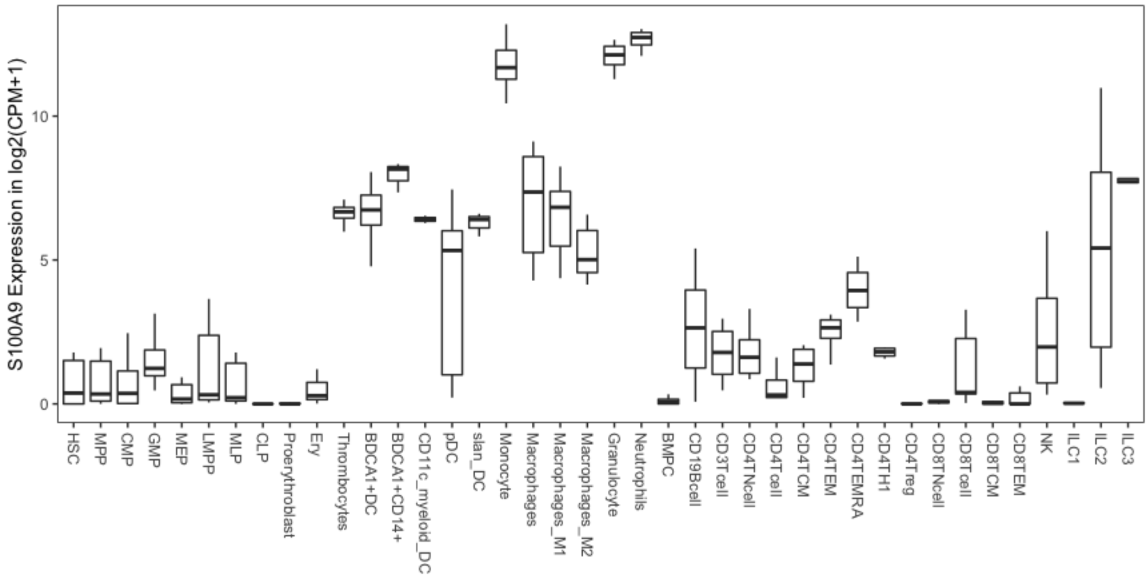

Figure S1: (A) RUNX3 and (B) S100A9 expression in the cell types of the *immunome*. Horizontal lines in the boxplot represent median expression values. CPM represents counts per million.

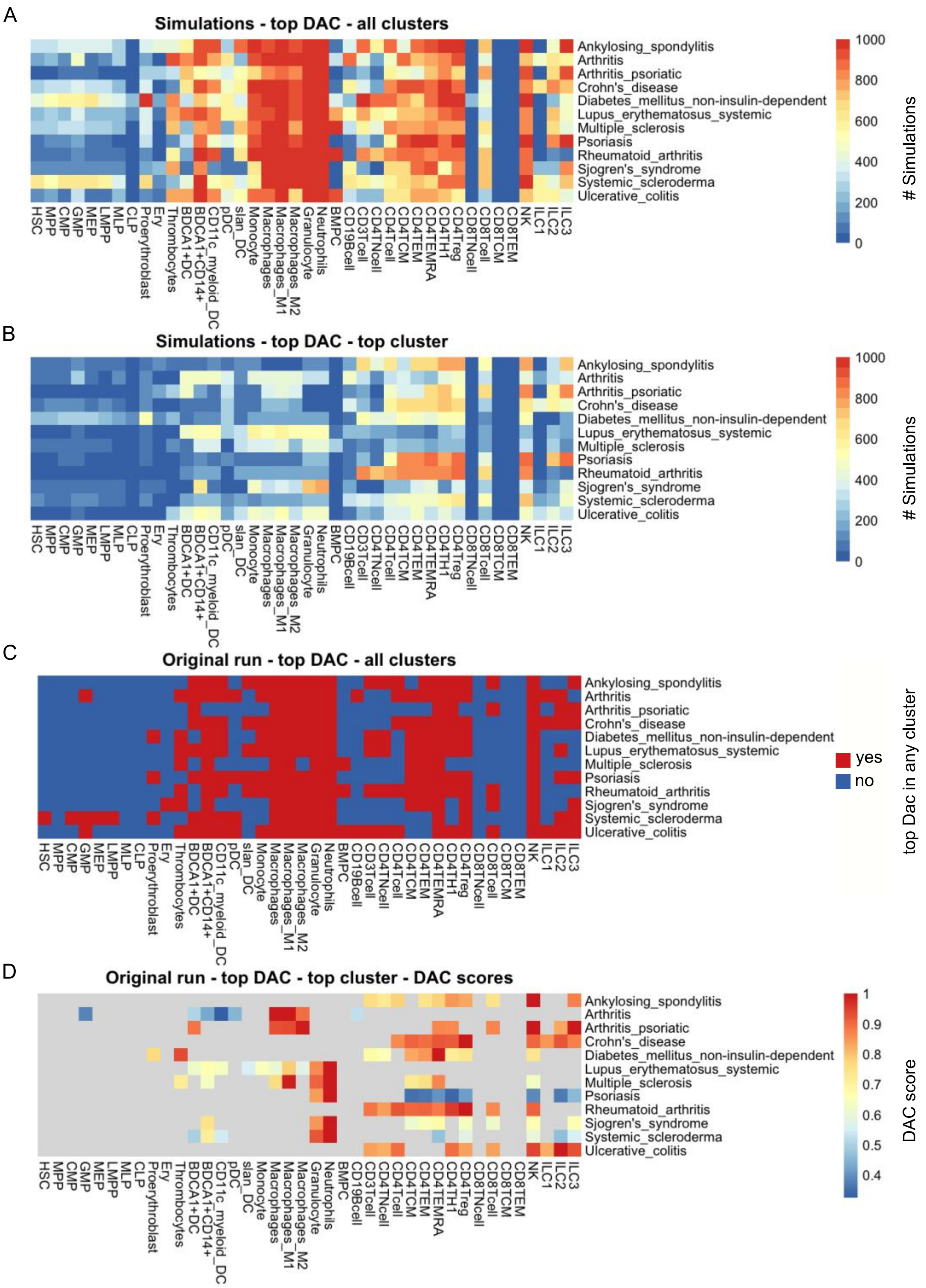

Figure S2: Consistency of top DACs. Heatmap of (**A**) top DACs from all clusters, (**B**) top DACs from top cluster – identified using 1000 jackknife simulations with 70% random subsampling of DAGs, (**C**) top DACs from all clusters of the original run, red grid represents if the cell type was a top DAC in any cluster, and (**D**) DAC score of the top DAC from the top cluster.

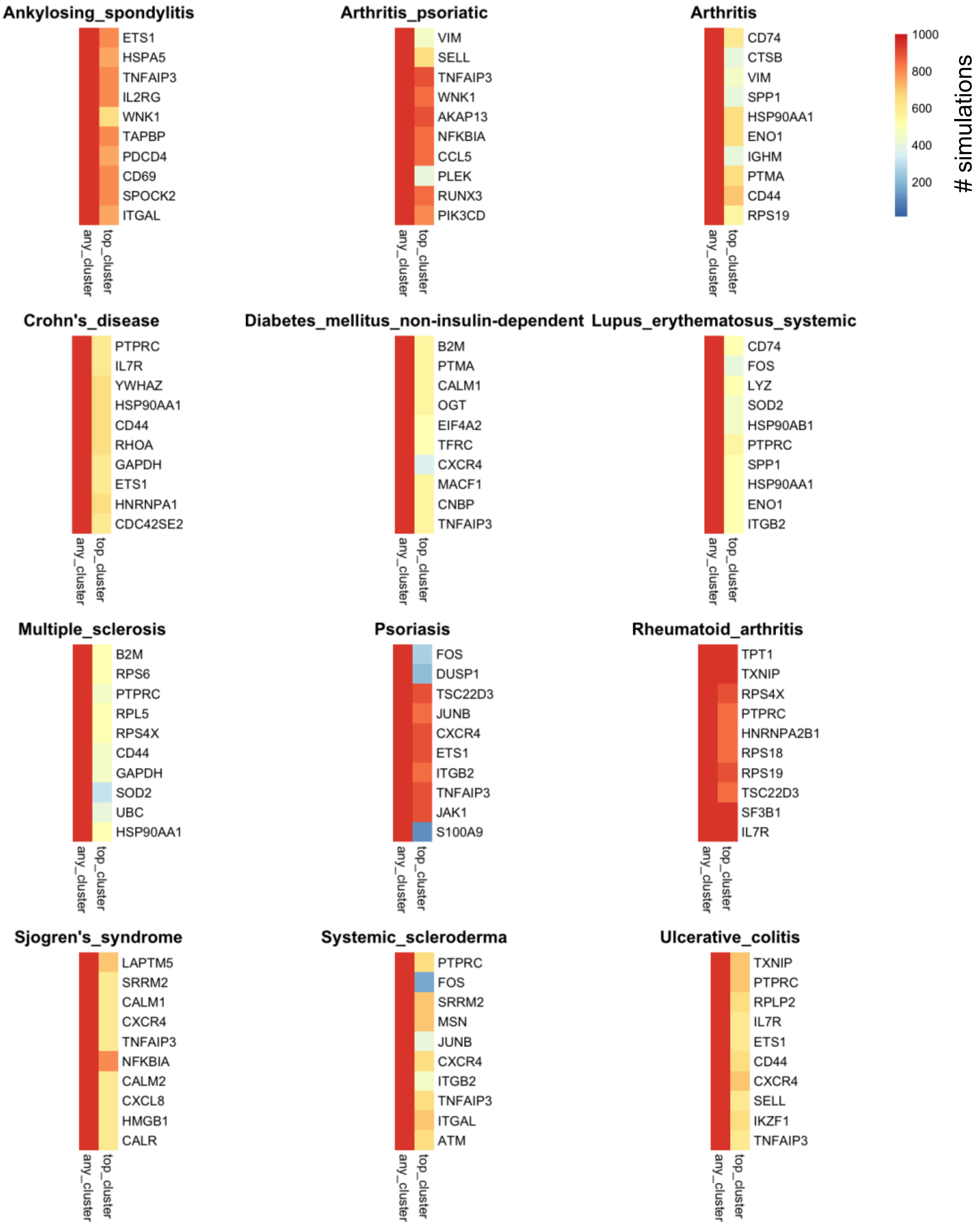

Figure S3: Consistency of top DAGs: Heatmap showing consitency of top 10 DAGs of the original run in the 1000 jackknife simulations with 70% random subsampling of cell types. Heatmap represents the numer of simulations in which, the top DAG was found in any cluster (column 1); was found in the top cluster (column 2).

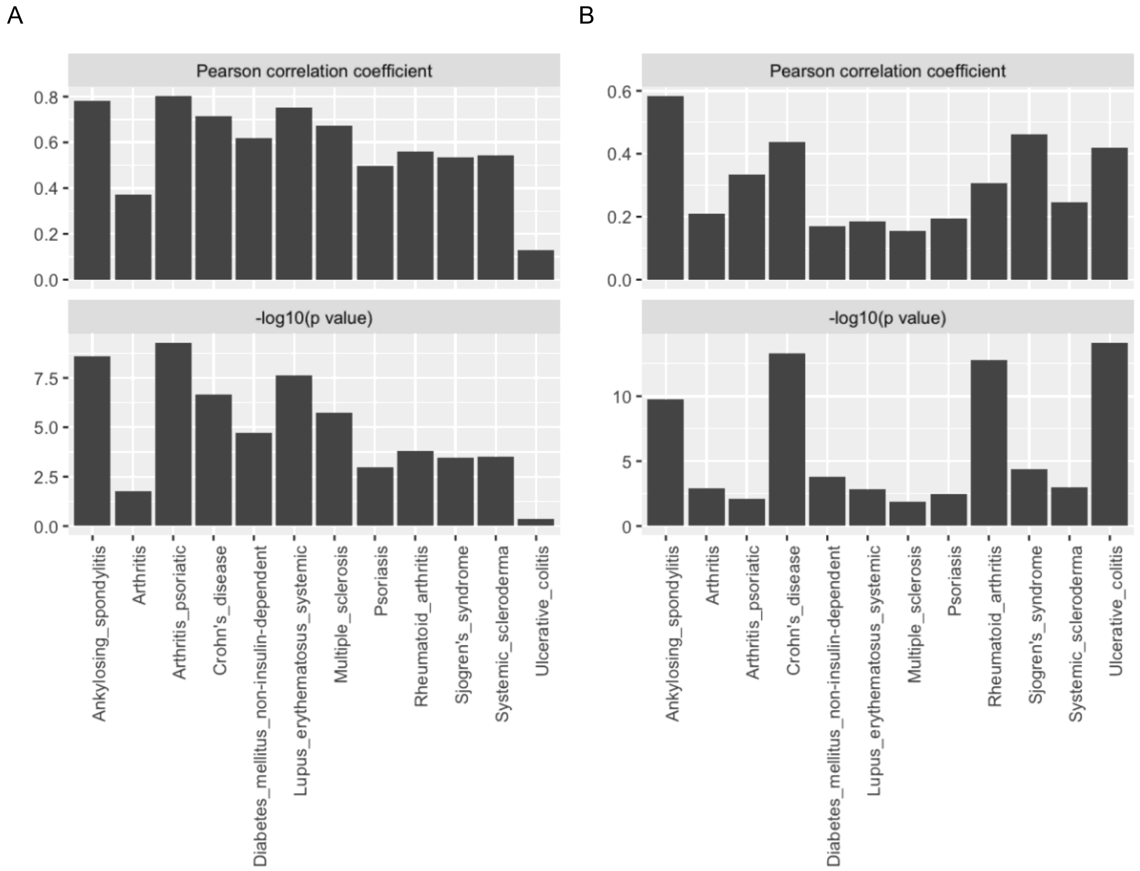

Figure S4: Pearson correlation coefficient and its p-value calculated between the 1000 jacknife simulations and (**A**) the DAC score, and (**B**) the DAG score, of the top cluster of the original run. p-values shown in negative log10 range. Negative log10 of 0.05 is 1.301.

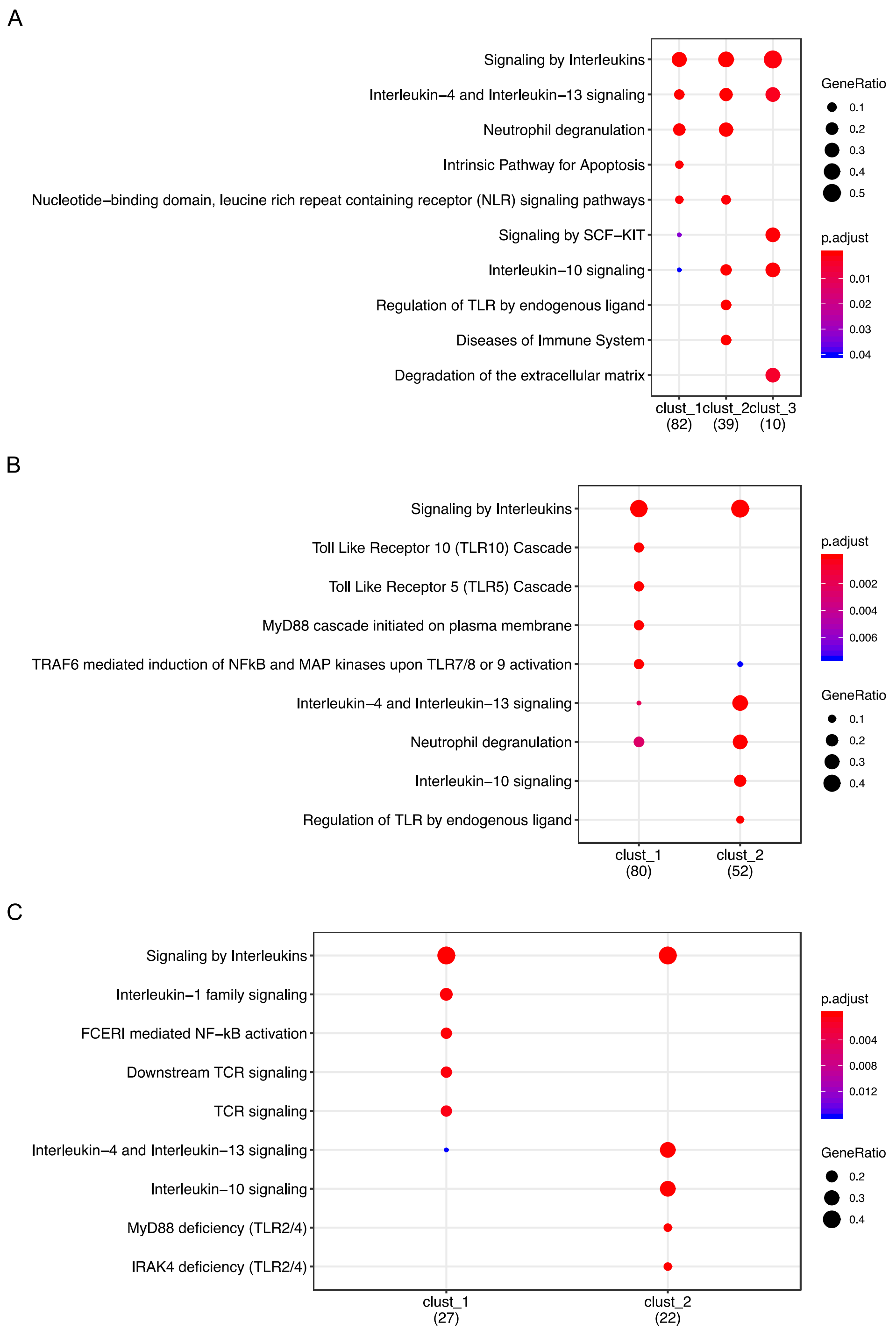

Figure S5: Pathway enrichment analysis of top DAGs of the common cell-gene networks between (**A**) CD and UC; (**B**). CD and RA; (**C**) AS and RA.

Table S1: GEO datasets and samples used to construct the immunome.

DATASET_ID	SAMPLE_ID	CELL_TYPE
GSE74246	SRR2753081	Ery
GSE74246	SRR2753084	Ery
GSE74246	SRR2753103	Ery
GSE51984	GSM1256817	Granulocyte
GSE51984	GSM1256818	Granulocyte
GSE51984	GSM1256819	Granulocyte
GSE51984	GSM1256820	Granulocyte
GSE51984	GSM1256821	Granulocyte
GSE62408	SRR1613937	Granulocyte
GSE60424	SRR1550986	Neutrophils
GSE60424	SRR1551047	Neutrophils
GSE60424	SRR1551054	Neutrophils
GSE60424	SRR1551068	Neutrophils
GSE81443	GSM2171212	BMPC
GSE81443	GSM2171213	BMPC
GSE81443	GSM2171214	BMPC
GSE81443	GSM2197431	BMPC
GSE81443	GSM2197432	BMPC
GSE81443	GSM2197433	BMPC
GSE51984	GSM1256812	CD19Bcell
GSE51984	GSM1256813	CD19Bcell
GSE51984	GSM1256814	CD19Bcell
GSE51984	GSM1256815	CD19Bcell
GSE51984	GSM1256816	CD19Bcell
GSE60424	SRR1550988	CD19Bcell
GSE60424	SRR1551049	CD19Bcell
GSE60424	SRR1551056	CD19Bcell
GSE60424	SRR1551070	CD19Bcell
GSE62408	SRR1613932	CD19Bcell
GSE64655	SRR1740034	CD19Bcell
GSE64655	SRR1740062	CD19Bcell
GSE66117	SRR1812751	CD19Bcell
GSE66117	SRR1812752	CD19Bcell
GSE66117	SRR1812753	CD19Bcell
GSE51984	GSM1256828	CD3Tcell
GSE51984	GSM1256829	CD3Tcell
GSE51984	GSM1256830	CD3Tcell
GSE51984	GSM1256831	CD3Tcell
GSE51984	GSM1256832	CD3Tcell
GSE64655	SRR1740058	CD3Tcell
GSE64655	SRR1740086	CD3Tcell

GSE107981	SRR6368708	CD4TNcell
GSE107981	SRR6368709	CD4TNcell
GSE107981	SRR6368710	CD4TNcell
GSE107981	SRR6368711	CD4TNcell
GSE58596	SRR1422906	CD4TNcell
GSE58596	SRR1422907	CD4TNcell
GSE58596	SRR1422908	CD4TNcell
GSE58596	SRR1422909	CD4TNcell
GSE62408	SRR1613935	CD4TNcell
GSE85294	SRR4011048	CD4TNcell
GSE85294	SRR4011051	CD4TNcell
GSE85294	SRR4011054	CD4TNcell
GSE97863	GSM2579315	CD4TNcell
GSE60424	SRR1550989	CD4Tcell
GSE60424	SRR1551050	CD4Tcell
GSE60424	SRR1551057	CD4Tcell
GSE60424	SRR1551071	CD4Tcell
GSE74246	SRR2753074	CD4Tcell
GSE74246	SRR2753078	CD4Tcell
GSE74246	SRR2753088	CD4Tcell
GSE74246	SRR2753112	CD4Tcell
GSE85294	SRR4011049	CD4TCM
GSE85294	SRR4011052	CD4TCM
GSE85294	SRR4011055	CD4TCM
GSE97863	GSM2579316	CD4TCM
GSE97863	GSM2579320	CD4TCM
GSE97863	GSM2579324	CD4TCM
GSE97863	GSM2579328	CD4TCM
GSE97863	GSM2579332	CD4TCM
GSE85294	SRR4011050	CD4TEM
GSE85294	SRR4011053	CD4TEM
GSE85294	SRR4011056	CD4TEM
GSE97863	GSM2579317	CD4TEM
GSE97863	GSM2579321	CD4TEM
GSE97863	GSM2579325	CD4TEM
GSE97863	GSM2579329	CD4TEM
GSE97863	GSM2579333	CD4TEM
GSE97863	GSM2579312	CD4TEMRA
GSE97863	GSM2579313	CD4TEMRA
GSE97863	GSM2579314	CD4TEMRA
GSE97863	GSM2579318	CD4TEMRA
GSE97863	GSM2579322	CD4TEMRA
GSE97863	GSM2579326	CD4TEMRA
GSE97863	GSM2579330	CD4TEMRA

GSE97863	GSM2579334	CD4TEMRA
GSE107981	SRR6368712	CD4TH1
GSE107981	SRR6368713	CD4TH1
GSE107981	SRR6368714	CD4TH1
GSE107981	SRR6368715	CD4TH1
GSE86452	GSM2302912	CD4Treg
GSE86452	GSM2302915	CD4Treg
GSE86452	GSM2302919	CD4Treg
GSE86452	GSM2302923	CD4Treg
GSE62408	SRR1613936	CD8TNcell
GSE63144	GSM1542163	CD8TNcell
GSE63144	GSM1542168	CD8TNcell
GSE63144	GSM1542173	CD8TNcell
GSE60424	SRR1550990	CD8Tcell
GSE60424	SRR1551051	CD8Tcell
GSE60424	SRR1551058	CD8Tcell
GSE60424	SRR1551072	CD8Tcell
GSE74246	SRR2753075	CD8Tcell
GSE74246	SRR2753079	CD8Tcell
GSE74246	SRR2753089	CD8Tcell
GSE74246	SRR2753113	CD8Tcell
GSE63144	GSM1542160	CD8TCM
GSE63144	GSM1542164	CD8TCM
GSE63144	GSM1542165	CD8TCM
GSE63144	GSM1542169	CD8TCM
GSE63144	GSM1542170	CD8TCM
GSE63144	GSM1542174	CD8TCM
GSE63144	GSM1542161	CD8TEM
GSE63144	GSM1542162	CD8TEM
GSE63144	GSM1542166	CD8TEM
GSE63144	GSM1542167	CD8TEM
GSE63144	GSM1542171	CD8TEM
GSE63144	GSM1542172	CD8TEM
GSE63144	GSM1542175	CD8TEM
GSE63144	GSM1542176	CD8TEM
GSE69596	SRR2052646	ILC1
GSE69596	SRR2052647	ILC1
GSE69596	SRR2052648	ILC1
GSE69596	SRR2052649	ILC1
GSE69596	SRR2052651	ILC1
GSE77088	SRR3111606	ILC2
GSE77088	SRR3111608	ILC2
GSE77088	SRR3111610	ILC2
GSE77088	SRR3111614	ILC2

GSE77088	SRR3111617	ILC2
GSE77088	SRR3111621	ILC2
GSE77088	SRR3111605	ILC3
GSE77088	SRR3111607	ILC3
GSE77088	SRR3111611	ILC3
GSE77088	SRR3111616	ILC3
GSE77088	SRR3111620	ILC3
GSE60424	SRR1550991	NK
GSE60424	SRR1551052	NK
GSE60424	SRR1551059	NK
GSE60424	SRR1551073	NK
GSE62408	SRR1613931	NK
GSE64655	SRR1740050	NK
GSE64655	SRR1740078	NK
GSE74246	SRR2753076	NK
GSE74246	SRR2753082	NK
GSE74246	SRR2753086	NK
GSE74246	SRR2753121	NK
GSE75042	SRR2919779	BDCA1+CD14+
GSE75042	SRR2919780	BDCA1+CD14+
GSE75042	SRR2919781	BDCA1+CD14+
GSE70106	SRR2072600	BDCA1+DC
GSE70106	SRR2072604	BDCA1+DC
GSE70106	SRR2072608	BDCA1+DC
GSE75042	SRR2919776	BDCA1+DC
GSE75042	SRR2919777	BDCA1+DC
GSE75042	SRR2919778	BDCA1+DC
GSE89442	SRR4841384	BDCA1+DC
GSE89442	SRR4841385	BDCA1+DC
GSE89442	SRR4841386	BDCA1+DC
GSE64655	SRR1740038	CD11c_myeloid_DC
GSE64655	SRR1740066	CD11c_myeloid_DC
GSE51984	GSM1256822	Monocyte
GSE51984	GSM1256823	Monocyte
GSE51984	GSM1256824	Monocyte
GSE51984	GSM1256825	Monocyte
GSE51984	GSM1256826	Monocyte
GSE58310	SRR1363132	Monocyte
GSE58310	SRR1363136	Monocyte
GSE58310	SRR1363140	Monocyte
GSE58310	SRR1363144	Monocyte
GSE60424	SRR1550987	Monocyte
GSE60424	SRR1551048	Monocyte
GSE60424	SRR1551055	Monocyte

GSE60424	SRR1551069	Monocyte
GSE64655	SRR1740042	Monocyte
GSE64655	SRR1740070	Monocyte
GSE70106	SRR2072602	Monocyte
GSE70106	SRR2072606	Monocyte
GSE70106	SRR2072610	Monocyte
GSE74246	SRR2753094	Monocyte
GSE74246	SRR2753100	Monocyte
GSE74246	SRR2753109	Monocyte
GSE74246	SRR2753119	Monocyte
GSE75042	SRR2919782	Monocyte
GSE75042	SRR2919783	Monocyte
GSE75042	SRR2919784	Monocyte
GSE75042	SRR2919785	Monocyte
GSE70106	SRR2072599	pDC
GSE70106	SRR2072603	pDC
GSE70106	SRR2072607	pDC
GSE79272	SRR3229088	pDC
GSE79272	SRR3229089	pDC
GSE79272	SRR3229090	pDC
GSE79272	SRR3229091	pDC
GSE79272	SRR3229092	pDC
GSE79272	SRR3229093	pDC
GSE89442	SRR4841393	pDC
GSE89442	SRR4841394	pDC
GSE89442	SRR4841395	pDC
GSE70106	SRR2072601	slan_DC
GSE70106	SRR2072605	slan_DC
GSE70106	SRR2072609	slan_DC
GSE55536	SRR2910663	Macrophages
GSE55536	SRR2939131	Macrophages
GSE55536	SRR2939132	Macrophages
GSE58310	SRR1363133	Macrophages
GSE58310	SRR1363137	Macrophages
GSE58310	SRR1363141	Macrophages
GSE58310	SRR1363145	Macrophages
GSE82227	SRR3623719	Macrophages
GSE82227	SRR3623728	Macrophages
GSE82227	SRR3623737	Macrophages
GSE82227	SRR3623746	Macrophages
GSE82227	SRR3623755	Macrophages
GSE36952	SRR452329	Macrophages_M1
GSE36952	SRR452330	Macrophages_M1
GSE55536	SRR2910664	Macrophages_M1

GSE55536	SRR2939133	Macrophages_M1
GSE55536	SRR2939134	Macrophages_M1
GSE82227	SRR3623721	Macrophages_M1
GSE82227	SRR3623730	Macrophages_M1
GSE82227	SRR3623739	Macrophages_M1
GSE82227	SRR3623748	Macrophages_M1
GSE82227	SRR3623757	Macrophages_M1
GSE36952	SRR452331	Macrophages_M2
GSE36952	SRR452332	Macrophages_M2
GSE36952	SRR452333	Macrophages_M2
GSE55536	SRR2910665	Macrophages_M2
GSE55536	SRR2939135	Macrophages_M2
GSE55536	SRR2939136	Macrophages_M2
GSE74246	SRR2753080	CLP
GSE74246	SRR2753083	CLP
GSE74246	SRR2753102	CLP
E-MTAB-5456	ERR1816826	CMP
E-MTAB-5456	ERR1816827	CMP
E-MTAB-5456	ERR1816828	CMP
E-MTAB-5456	ERR1816829	CMP
GSE74246	SRR2753090	CMP
GSE74246	SRR2753096	CMP
GSE74246	SRR2753104	CMP
GSE74246	SRR2753114	CMP
GSE76234	SRR3039600	CMP
GSE76234	SRR3039601	CMP
GSE76234	SRR3039606	CMP
GSE76234	SRR3039607	CMP
GSE76234	SRR3039612	CMP
GSE76234	SRR3039618	CMP
E-MTAB-5456	ERR1816830	GMP
E-MTAB-5456	ERR1816831	GMP
E-MTAB-5456	ERR1816832	GMP
E-MTAB-5456	ERR1816833	GMP
GSE74246	SRR2753091	GMP
GSE74246	SRR2753097	GMP
GSE74246	SRR2753105	GMP
GSE74246	SRR2753115	GMP
GSE76234	SRR3039613	GMP
GSE76234	SRR3039619	GMP
E-MTAB-5456	ERR1816834	HSC
E-MTAB-5456	ERR1816835	HSC
E-MTAB-5456	ERR1816836	HSC
E-MTAB-5456	ERR1816837	HSC

GSE74246	SRR2753092	HSC
GSE74246	SRR2753098	HSC
GSE74246	SRR2753106	HSC
GSE74246	SRR2753116	HSC
GSE76234	SRR3039602	HSC
GSE76234	SRR3039608	HSC
E-MTAB-5456	ERR1816838	LMPP
E-MTAB-5456	ERR1816839	LMPP
E-MTAB-5456	ERR1816840	LMPP
E-MTAB-5456	ERR1816841	LMPP
GSE74246	SRR2753085	LMPP
GSE74246	SRR2753107	LMPP
GSE74246	SRR2753117	LMPP
E-MTAB-5456	ERR1816842	MEP
E-MTAB-5456	ERR1816843	MEP
E-MTAB-5456	ERR1816844	MEP
E-MTAB-5456	ERR1816845	MEP
GSE74246	SRR2753093	MEP
GSE74246	SRR2753099	MEP
GSE74246	SRR2753108	MEP
GSE74246	SRR2753118	MEP
GSE76234	SRR3039603	MEP
GSE76234	SRR3039609	MEP
GSE76234	SRR3039614	MEP
GSE76234	SRR3039615	MEP
GSE76234	SRR3039620	MEP
GSE76234	SRR3039621	MEP
E-MTAB-5456	ERR1816846	MLP
E-MTAB-5456	ERR1816847	MLP
E-MTAB-5456	ERR1816848	MLP
E-MTAB-5456	ERR1816849	MLP
GSE76234	SRR3039604	MLP
GSE76234	SRR3039610	MLP
E-MTAB-5456	ERR1816850	MPP
E-MTAB-5456	ERR1816851	MPP
E-MTAB-5456	ERR1816852	MPP
E-MTAB-5456	ERR1816853	MPP
GSE74246	SRR2753095	MPP
GSE74246	SRR2753101	MPP
GSE74246	SRR2753110	MPP
GSE74246	SRR2753120	MPP
GSE76234	SRR3039605	MPP
GSE76234	SRR3039611	MPP
GSE76234	SRR3039616	MPP

GSE76234	SRR3039617	MPP
GSE76234	SRR3039622	MPP
GSE76234	SRR3039623	MPP
GSE107218	GSM2862914	Proerythroblast
GSE107218	GSM2862915	Proerythroblast
GSE107218	GSM2862916	Proerythroblast
GSE68086	GSM1662648	Thrombocytes
GSE68086	SRR1982696	Thrombocytes
GSE68086	SRR1982697	Thrombocytes
GSE68086	SRR1982700	Thrombocytes
